# Examining the Relationship between Cognitive Function and Sleep Disturbance in Pregnancy and Postpartum

**DOI:** 10.1192/j.eurpsy.2026.12092

**Published:** 2026-07-31

**Authors:** Y. Schmitt, L. Ospina, M. MccLure, M. M. Perez-Rodriguez

**Affiliations:** Psychiatry, Icahn School of Medicine at Mount Sinai, New York, United States

## Abstract

**Introduction:**

The impact of sleep disturbances on cognitive function is well-established in the general population. However, during the psychiatric high-risk periods of pregnancy and postpartum, characterized by substantial sleep disturbances, the impact of sleep on cognition is understudied. Some studies have found that the connection between sleep disturbance and cognitive function may be strongest among individuals experiencing depressive symptoms.

**Objectives:**

We administered a comprehensive cognitive battery in pregnant and postpartum individuals and examined its relationship with sleep disturbance in the third trimester of pregnancy and at three months postpartum.

**Methods:**

Participants completed the Cambridge Neuropsychological Test Automated Battery (CANTAB) in the 3rd trimester (N=274) and at 3 months postpartum (N=203). Tasks assessed executive functioning (One Touch Stockings of Cambridge, Cambridge Gambling Task), memory (Paired Associates Learning, Spatial Working Memory, Delayed Matching to Sample, Pattern Recognition Memory), and attention (Rapid Visual Information Processing). A subset also completed the PHQ-9, a screening measure which assesses sleep difficulty, and the Edinburgh Postnatal Depression Scale (EPDS). Sleep disturbance was defined as a score >0 on PHQ-9 item 3. A cross-sectional analysis was performed at each time point correlating the PHQ-9 item with the 7 individual cognitive task z-scores and composite domain scores using Spearman’s rank correlation. Additionally, the correlation between change in sleep items and z-scores within participants at each time point was examined using Spearman’s rank correlation. Finally, we examined the prevalence of mild cognitive impairment, defined as a z-score ≤−1, across both timepoints and its association PHQ-9 item 3. Analyses were performed at three levels: (1) unadjusted, (2) stratified by EPDS total (<10 vs ≥10), and (3) adjusted for EPDS score using rank-based regression.

**Results:**

During pregnancy, **51.7%** had a sleep disturbance on PHQ-9 item 3 (N=**149**). At 3 months postpartum **30.2%** had a sleep disturbance on PHQ-9 item 3 (N=**129**). Greater increases in sleep disturbance were associated with greater decreases in quality of decision making on the Cambridge Gambling Task between pregnancy and postpartum (Estimate = **-0.45**, P = **0.01**; N = **62**), after adjusting for EDPS depression scores. In unadjusted and EPDS stratified analysis, there were no significant cross-sectional or longitudinal associations between PHQ-9 sleep scores and cognition.

**Conclusions:**

We found an association between increased sleep disturbance between pre-and post-partum and quality of decision making when controlling for depression. Further research is needed to explore potential mediating factors and to use more fine-grained measures of sleep quality and duration.

**Disclosure of Interest:**

Y. Schmitt: None Declared, L. Ospina: None Declared, M. MccLure: None Declared, M. M. Perez-Rodriguez Grant / Research support from: Principal Investigator of Grant to Mount Sinai from Neurocrine Biosciences (unrelated to this presentation), Consultant of: Consultant with Neurocrine Biosciences and Mitsubishi Tanabe Pharma Corporation (unrelated to this presentation).

